# Identification of functional genetic variants associated with prostate cancer through analysis of genome-wide genetic and epigenetic datasets

**DOI:** 10.1186/gb-2011-12-s1-p24

**Published:** 2011-09-19

**Authors:** McAnthony Tarway, Wei Tang, Ludmila Prokunina-Olsson

**Affiliations:** 1 Laboratory of Translational Genomics, Division of Cancer Epidemiology and Genetics, National Cancer Institute, National Institutes of Health, Bethesda, MD 20892, USA

## Background

Recent genome-wide association studies (GWAS) have identified multiple genetic variants associated with the risk of developing prostate cancer (PrCa). At least ten PrCa-associated single nucleotide polymorphisms (SNPs) are located within a gene-poor region on chromosome 8q24, but the functional mechanisms of each of these variants remain unknown. Normal prostate development, as well as tumor initiation and progression, greatly depends on the androgen receptor (AR) and its ligands, testosterone and 5α-dihydrotestosterone. We hypothesized that genetic variants associated with PrCa risk might be important owing to their effects on AR-binding sites.

## Methods and results

We comprehensively explored 11 PrCa GWAS published as of July 2011 in the National Human Genome Research Institute’s GWAS database [1] and in PubMed [2]. We selected ten SNPs from the 8q24 region that were significantly and consistently associated with PrCa in Caucasian datasets (*P* < 5 × 10^–7^). By querying the CEU 1000 Genomes Project panel, we generated a list of 224 SNPs in high linkage disequilibrium (*r*^2^ > 0.8) with the ten selected GWAS SNPs. Of all of the SNPs on this list, six variants were located in the regions identified as AR-binding sites, based on AR chromatin immunoprecipitation (ChIP)-Seq data from the University of California, Santa Cruz’s genome browser [3]. To test for differential binding of AR to alleles of the six SNPs, we developed a protocol for quantitative multiplex allele-specific ChIP (AS-ChIP) assays. Confirmatory AS-ChIP with AR-specific antibodies in the LNCaP cell line showed that five of these SNPs were heterozygous in the LNCaP cell line, and four of them showed statistically significant allele-specific differences in AR binding (*P*-value range = 0.0005 to 0.04, based on four biological replicates of AS-ChIP).

## Conclusions

Our data suggest that some of the PrCa-associated SNPs within the 8q24 region might create or disrupt binding sites for AR, thereby affecting important regulatory networks in normal and cancerous prostate tissue.
